# Energy-Efficient Resource Allocation Based on Deep Q-Network in V2V Communications

**DOI:** 10.3390/s23031295

**Published:** 2023-01-23

**Authors:** Donghee Han, Jaewoo So

**Affiliations:** Department of Electronic Engineering, Sogang University, Seoul 04107, Republic of Korea

**Keywords:** vehicular communications, deep reinforcement learning, deep Q-network, resource allocation, energy efficiency

## Abstract

Recently, with the development of autonomous driving technology, vehicle-to-everything (V2X) communication technology that provides a wireless connection between vehicles, pedestrians, and roadside base stations has gained significant attention. Vehicle-to-vehicle (V2V) communication should provide low-latency and highly reliable services through direct communication between vehicles, improving safety. In particular, as the number of vehicles increases, efficient radio resource management becomes more important. In this paper, we propose a deep reinforcement learning (DRL)-based decentralized resource allocation scheme in the V2X communication network in which the radio resources are shared between the V2V and vehicle-to-infrastructure (V2I) networks. Here, a deep Q-network (DQN) is utilized to find the resource blocks and transmit power of vehicles in the V2V network to maximize the sum rate of the V2I and V2V links while reducing the power consumption and latency of V2V links. The DQN also uses the channel state information, the signal-to-interference-plus-noise ratio (SINR) of V2I and V2V links, and the latency constraints of vehicles to find the optimal resource allocation scheme. The proposed DQN-based resource allocation scheme ensures energy-efficient transmissions that satisfy the latency constraints for V2V links while reducing the interference of the V2V network to the V2I network. We evaluate the performance of the proposed scheme in terms of the sum rate of the V2X network, the average power consumption of V2V links, and the average outage probability of V2V links using a case study in Manhattan with nine blocks of 3GPP TR 36.885. The simulation results show that the proposed scheme greatly reduces the transmit power of V2V links when compared to the conventional reinforcement learning-based resource allocation scheme without sacrificing the sum rate of the V2X network or the outage probability of V2V links.

## 1. Introduction

Today, with the development of autonomous driving technologies, vehicular communication technologies are receiving significant attention from both the industry and academia [1,2]. The 3GPP has recently designed a new radio (NR) sidelink to support direct vehicle-to-vehicle (V2V) communication without the help of a base station (BS) in a low-latency, high-throughput, and high-connection-density network [1,3,4]. V2V networks require ultra-reliable and low-latency communication (URLLC) services for use cases that demand certain safety features, such as autonomous driving systems that send and receive warning messages to and from nearby vehicles, even as the number of vehicles increases [5]. Therefore, it is important to manage radio resources efficiently to satisfy the quality of service (QoS) of vehicles in the V2V network.

Radio resource management is often formulated as a set of combined optimizations used to find the optimal solution of an objective problem, which is generally an NP-hard problem. In recent years, machine learning has been successfully applied in a wide range of areas, resulting in significant performance improvements. In particular, reinforcement learning (RL) has shown its superiority in solving the resource allocation problems in communications [6,7]. Resource allocation can be dived into three categories according to which layer of the OSI 7-layer model performs [8,9,10]. The first category is bandwidth allocation at the network layer, which aims to provide call-level QoS guarantees. The second category is the allocation of resource blocks (RBs) at the link layer. The link layer determines which RB the transmitter will use, on the basis of the channel state measured in the physical layer. The third category is the joint RB and power allocation at the cross-layer between the link layer and the physical layer [9,10]. In this paper, we focus on the resource allocation of the RB and the transmit power of V2V links at both the link layer and the physical layer. Resource allocation is based on the channel state information (CSI), i.e., the signal-to-interference-plus-noise ratio (SINR). We propose a deep Q-network (DQN)-based spectrum and power allocation scheme for energy-efficient V2V communications while maximizing the sum rate of the V2I and V2V links. The proposed Q-network uses the CSI of V2I and V2V links and the latency constraints of vehicles to find the optimal RB and transmit power of the V2V links. The contributions of this paper are as follows: First, we developed a decentralized resource allocation problem that incorporates the power consumption as well as the latency of V2V links while increasing the sum rate of the V2I and V2V links. Second, we developed a DQN model to solve the resource allocation problem, where the reward function includes the power consumption and latency conditions represented as penalties. Third, the simulation results show that the DQN-based energy-efficient resource allocation scheme greatly reduces overall power consumption in comparison with the conventional RL-based scheme without sacrificing the sum rate and latency requirements of V2V links.

The rest of the paper is organized as follows. Section 2 describes the system model. Section 3 presents a DQN-based resource allocation scheme, where the state, action, and reward functions of reinforcement learning (RL) are described in detail. Section 4 shows the simulation results in a case study of Manhattan. Finally, Section 5 concludes the paper.

## 2. Related Work

The resource allocation mechanism in vehicle-to-everything (V2X) communications has been studied in various ways. The authors of [11] introduced the deep reinforcement learning (DRL)-based resource allocation scheme and showed experimental results for both unicast and broadcast scenarios. They designed a reward function to ensure the latency constraints of the V2V links were satisfied. The authors of [12] proposed a QoS-aware resource allocation scheme based on the DRL framework in V2X communications, where they took QoS parameters such as the priority of V2V messages into consideration. The proposed scheme of [12] aims to maximize the sum rate of vehicle-to-infrastructure (V2I) links while satisfying the latency constraints of V2V links. The authors of [13] developed a power allocation problem in the cellular device-to-device (D2D)-based V2X communication network and mathematically solved the problem. They showed that the proposed power allocation scheme outperforms the existing algorithms in terms of power consumption. The authors of [14] developed a multi-agent RL (MARL)-based resource allocation for V2V links in the spectrum-sharing V2X network. They aimed to maximize the capacity of V2I links while also improving the reliability of the payload delivery in V2V links. They showed the MADRL-based resource allocation is efficient for the V2I and V2V network collaboration although decisions are made locally and distributed at each V2V transmitter. The authors of [15] proposed a MARL-based resource allocation scheme in order to maximize the sum rate of V2I links while satisfying the latency and reliability requirements of V2V links. In this work, they developed individual double-dueling deep recurrent Q-networks (D3RQN), where they used interference power measurements instead of the conventional CSI under the assumption that it is difficult to acquire the perfect CSI in the vehicular network. They showed that the proposed MARL-based resource allocation jointly adjusts the sub-channel and transmit power using only local interference measurements without inter-agent communication. Some studies have focused on the energy consumption in V2X networks. The authors of [16] developed an energy efficiency problem in an NR V2X network, where energy efficiency is defined as the ratio of the sum rate to power consumption. They proposed a heuristic algorithm of traffic-density-based random selection to solve the developed mixed-integer problem. The authors of [17] also developed an energy efficiency problem of vehicle users while considering the QoS requirement of cellular users in the cellular network underlying V2V communications. They transformed the latency constraint into the constraint of the queue length and solved the virtual queue problem based on the Lyapunov optimization. In V2X networks, the energy consumption of V2I links as well as V2V links is important. Some studies have focused on optimizing energy consumption across the entire wireless access network [18,19,20]. The authors of [18] proposed an energy-efficient resource management scheme based on the transmit power scaling and on/off switching of base stations. The authors of [19] formulated an optimization problem for the energy consumption of a wireless location area network (WLAN) by adjusting the transmit power and turning access stations on and off based on realistic traffic patterns. They proposed integer linear programming (ILP) optimization models and heuristic algorithms to minimize the energy consumption of the network. The authors of [20] developed an ILP model for energy saving of wireless access networks, and also developed a heuristic algorithm based on a greedy method to cope with the computational complexity of the ILP model. 

Recently, graph-based deep learning solutions have been proposed for resource allocation in communication networks [7,21,22,23,24]. Graph neural networks (GNNs) have achieved some success in solving resource allocation problems in various communication networks, e.g., wireless networks, wired networks, and software-defined networks, because of their abilities to learn to capture the dependencies of graphs and to learn non-Euclidean structure data [21]. The authors of [22] presented a comprehensive review and analysis of graph-based resource allocation methods in cellular, device-to-device, and cognitive radio networks. Here, they classified the graph-based resource allocation methods in terms of graph models, tasks solved via graphs, graph formulation, and optimization methods. The authors of [23] proposed a heterogeneous bipartite GNN (HBGNN) to solve the joint user association and power allocation problem in heterogeneous ultra-dense networks (HUDNs). They modeled the downlink of the HUDN as a heterogeneous bipartite graph and compared the performance of the proposed HBGNN with the fully connected neural network and the convolutional neural network (CNN). However, the HBGNN requires supervised learning, unlike RL. The authors of [24] developed a graph convolutional network (GCN)-based DRL framework to perform joint channel selection and power adaptation in the underlying cognitive radio networks, maximizing the data rate of secondary users while maintaining the level of interference to primary users. They modeled the environment of the cognitive radio network as a dynamic graph and adopted a DRL to explore the optimal resource allocation strategy. However, the work of [24] did not take the energy efficiency and the latency constraints of the secondary users into account. The authors of [7] presented a GNN-augmented RL method to perform spectrum allocation for vehicular networks. They expressed the V2V network as a graph and exploited RL to perform resource allocation. The deep Q-network was developed to select the spectrum for each V2V pair.

Deep learning technologies for Internet of vehicle (IoV) networks have been studied previously [25,26,27,28,29]. The authors of [25] discussed deep learning applications for security and collision prediction in the internet of vehicle (IoV) networks, and they proposed a DRL-based resource allocation method to enhance multiple QoS requirements, such as latency and suitable data rate requirements. They introduced an actor–critic framework to achieve an intelligent resource allocation in the IoV network. The authors of [26] discussed deep learning techniques to enhance the performance of the overall IoV system. They addressed various learning networks, e.g., CNN, recurrent neural networks, DRL, classification, clustering, and regression. The authors of [27] presented a comprehensive review and analysis of machine learning technologies for IoV applications, e.g., energy- and buffer-aware optimization, edge caching, intelligent decisions for network scheduling and adaptation, intelligent autonomous driving, etc. The authors of [28] presented a comprehensive review of resource allocation and management for the IoV over 5G radio access networks. They described learning-based resource allocation approaches to improve the QoS and quality of experience in distributed and cloud-computing resource allocation schemes, along with big data resource allocation. The authors of [29] conducted a critical review and analysis of machine learning models used to resolve the challenge in IoV applications. Moreover, they proposed a Markov decision-process-based, edge-computing offloading model and evaluated its performance in terms of its power consumption and task latency.

Moreover, vehicular edge computing (VEC) technologies have been studied to dynamically manage computing resources, caching, and networking [30]. The authors of [31] proposed a generic approach to improve the performance of application outsourcing in the caching-assisted VEC. They mathematically showed that application caching can optimize the average response time while satisfying the long-term energy consumption constraint. The authors of [32] addressed route planning in a navigation system that finds an optimal route from the source to the target location. They proposed a real-time cache-aided route planning system based on mobile edge computing with the aim of reducing the communication delay between the access network and the remote central server and the computational time of route planning queries. The authors of [33] proposed a caching-enabled VEC scheme for jointly optimizing task caching and computation offloading in a VEC system; task caching was shown to reduce response latency but increase energy consumption. They then formulated an optimization problem that minimizes the weighted sum of the service time and energy consumption in the caching-assisted VEC system and used a genetic algorithm to solve the problem. The authors of [34] presented a comprehensive review and analysis of the vehicle routing problem (VRP). They mainly reviewed machine learning-assisted VRP modeling and optimization approaches.

## 3. System Model

**Figure 1:**
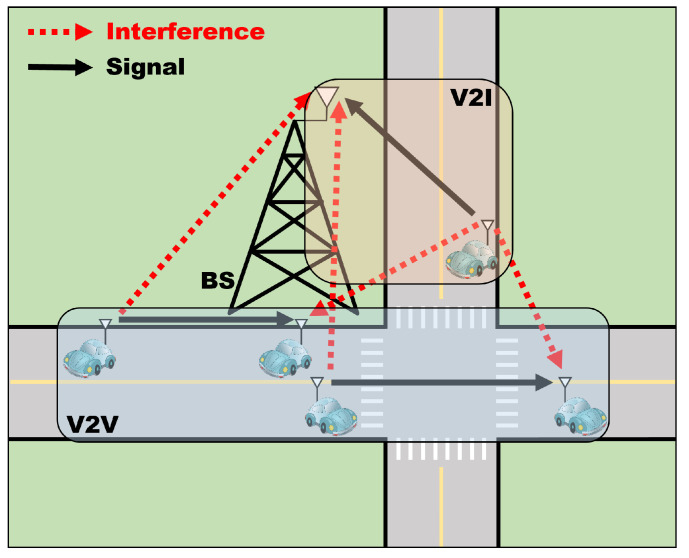
A system model.

We consider a V2X network consisting of a V2I network and a V2V network as shown in Figure 1. We focus on the uplink in the V2I network, where there are *L* V2I links denoted by L={1,2,…,L}. In the V2V network, there are *K* V2V links denoted by K={1,2,…,K}. In the V2I network, the spectrum is orthogonally allocated to the vehicles, where the number of orthogonal RBs is $N_{\mathrm{RB}}$. However, the V2V links share the resources, $N_{\mathrm{RB}}$, of the V2I network.

In the V2I network, the received SINR and capacity of the *l*th V2I link are represented as follows:

(1) $$\begin{array}{ccc} {\mathrm{SIN}R}_{l} & = & \frac{P_{l}^{V2I}h_{l}}{\sigma^{2}+\sum_{k\in K}\mu_{k,l}p_{k}h_{k}} \end{array}$$

(2) $$\begin{array}{ccc} C_{l} & = & W\log\left(1+{\mathrm{SIN}R}_{l}\right)\;\;[\mathrm{bits}/\mathrm{second}], \end{array}$$

where $P_{l}^{V2I}$ is the transmit power of the vehicle and $h_{l}$ is the channel power gain in the *l*th V2I link. Additionally, $p_{k}$ is the transmit power of the *k*th V2V link, $h_{k}$ is the channel power gain from the transmitter of the *k* V2V link to the base station, $\sigma^{2}$ is the noise power, and *W* is the bandwidth. The indicator function, $\mu_{k,l}$, denotes whether the resource is shared between the *k*th V2V link and the *l*th V2I link. That is, if the *k*th V2V link shares the RB of the *l*th V2I link, $\mu_{k,l}=1$; otherwise, $\mu_{k,l}=0$.

In the V2V network, the received SINR and capacity of the *k*th V2V link are represented as follows:

(3) $$\begin{array}{ccc} {\mathrm{SIN}R}_{k} & = & \frac{p_{k}g_{k}}{\sigma^{2}+I_{k}^{V2V}+I_{k}^{V2I}} \end{array}$$

(4) $$\begin{array}{ccc} I_{k}^{V2I} & = & \sum_{l\in L}\mu_{k,l}P_{l}^{V2I}g_{l,k} \end{array}$$

(5) $$\begin{array}{ccc} I_{k}^{V2V} & = & \sum_{l\in L}\sum_{j\in K,j\neq k}\mu_{k,l}\mu_{j,l}p_{j}g_{j,k} \end{array}$$

(6) $$\begin{array}{ccc} C_{k} & & =W\log\left(1+{\mathrm{SIN}R}_{k}\right)\;\;[\mathrm{bits}/\mathrm{second}], \end{array}$$

where $p_{k}$ is the transmit power of the vehicle and $g_{k}$ is the channel power gain in the *k*th V2V link. Additionally, $I_{k}^{V2I}$ is the interference from the V2I link sharing the RB of the *k*th V2V link, $I_{k}^{V2V}$ is the interference from the V2V link sharing the RB of the *k*th V2V link, and $g_{l,k}$ is the channel power gain from the transmitter of the *l*th V2I link to the receiver of the *k*th V2V link. The indicator function, $\mu_{j,l}$, denotes whether the resource is shared between the *j*th V2V link and the *l*th V2I link. That is, if the *j*th V2V link shares the RB of the *l*th V2I link, $\mu_{j,l}=1$; otherwise, $\mu_{j,l}=0$.

In order for the BS to know the channel state of the V2V links, each receiver of the V2V link reports its CSI to the BS, which results in a large signaling overhead. Hence, we assume that the BS does not know the CSI of the V2V links. The BS independently controls the resource allocation of the V2I links without considering the channel state of the V2V links. Consequently, vehicles on the V2V link individually select the RB and determine the transmit power based on the locally observed channel information. Here, the locally observed channel information in the V2V link consists of the following: the CSI of the V2I link, the interference power observed in the previous time slot, the instantaneous CSI of the V2V link, and the information on the RB selected by nearby vehicles.

Our objective is to maximize the sum rate of the V2I links while increasing the probability of meeting the latency constraint of the V2V links by controlling the selection of RB and the transmit power of each V2V link. However, finding the optimal allocations of the RB and transmit power is an NP-hard problem. Hence, we propose a DQN-based approach to solve the resource allocation problem.

## 4. Deep Q-Network for Energy-Efficient Resource Allocation

### 4.1. Reinforcement Learning

In RL, an agent observes a state in an environment that satisfies the Markov decision process (MDP). Then, the optimal action is selected according to the given policy. Depending on the selected action, the agent interacts with the environment, receives a reward from the environment, and transitions to the next state.

**Figure 2:**
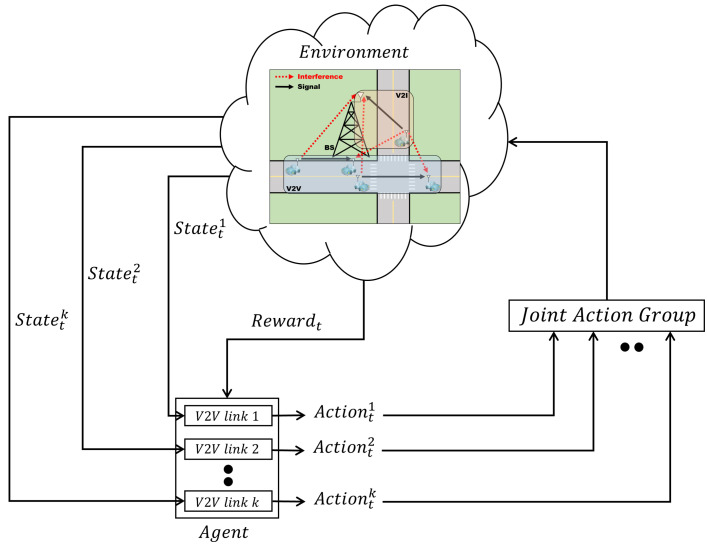
The structure of the RL for the vehicular network.

The goal of RL is to maximize the expected return value after the episode ends. The return formula is given as follows:

(7) $$R_{t:T}=r_{t}+\gamma r_{t+1}+\gamma^{2}r_{t+2}+\ldots +\gamma^{T}r_{t+T-1},$$

where $r_{t}$ denotes the reward obtained immediately at time *t*, *T* is the time step, and γ denotes the discount factor. The structure of our RL is shown in Figure 2. The agent observes the state of the environment at the time (*t*) and selects the best action according to the given policy. When the agent selects an action for all V2V links, the actions are stored in the joint action group and interact with the environment at the same time, and the agent receives a reward. In our system model, we develop the RL with the following parameters:

1. *State space*: We use the following state, similar to the unicast scenario of [11].
  (8) $$s_{t}=\left\{H_{t},I_{t-1},G_{t},N_{t-1},U_{t},L_{t}\right\},$$
  where $H_{t}$ is the CSI of V2I links at time *t*; $I_{t-1}$ is the interference power to the link at time t−1; $G_{t}$ is the instantaneous CSI of the corresponding V2V link at time *t*; $N_{t-1}$ is the information of RBs selected by surrounding vehicles at time t−1; $U_{t}$ is the time remaining to satisfy the latency constraints at time *t*; and $L_{t}$ is the remaining data to be received from the transmitter of the V2V link at time *t*. $H_{t}$, $I_{t-1}$, $G_{t}$, and $N_{t-1}$ are vectors containing the state information of the corresponding RBs, and $U_{t}$ and $L_{t}$ are scalar values that are the time remaining to satisfy the latency constraints and the remaining data, respectively. Therefore, the dimension of the state space is given by $D_{\mathrm{state}}=\left(4\times N_{\mathrm{RB}}\right)+2$.
2. *Action space*: The action determines the transmit power and the allocation of RBs. Hence, the dimensions of the action space are given by $D_{\mathrm{action}}=N_{\mathrm{pwr}}\times N_{\mathrm{RB}}$, where $N_{\mathrm{pwr}}$ is the number of transmit power levels in the V2V link and $N_{\mathrm{RB}}$ is the number of RBs.
3. *Reward*: We formulate the following reward function taking two penalties into account, the transmission time and the power consumption:
  (9) $$r_{t}=\lambda_{V2I}\sum_{l\in \;L}C_{l}+\lambda_{V2V}\sum_{k\in K}C_{k}-\lambda_{\mathrm{latency}}\left(T_{0}-U_{t}\right)-\lambda_{\mathrm{pwr}}\frac{1}{K}\sum_{k\in K}\frac{p_{k}}{p_{\max}},$$
  where $T_{0}$ is the maximum tolerable latency, and therefore, $\left(T_{0}-U_{t}\right)$ means the transmission time. Moreover, $p_{\max}$ is the maximum transmit power in the V2V link. $\lambda_{V2I}$ and $\lambda_{V2V}$ represent the weight for the sum rate of the V2I links and the sum rate of the V2V links, respectively. $\lambda_{\mathrm{latency}}$ and $\lambda_{\mathrm{pwr}}$ represent the weight of the penalty according to an increase in the transmission time and the penalty according to an increase in the transmit power, respectively. As the sum rate of the V2I or V2V links increases, a positive factor is added. However, as the transmission time or power consumption increases, a negative factor is added.

### 4.2. Deep Q-Network

A frequently used framework in the RL is a DQN [35,36,37,38]. The DQN framework is a structure that includes a Q-network consisting of a deep neural network (DNN) in the Q-learning structure. In order to train the Q-network in the DQN framework, several learning methods need to be applied [39].

In Q-learning, the Q-value means the expected return when reaching the terminal state from the state observed in time *t*, as follows:

(10) $$Q\left(s,a\right)=E\left[R_{t:T}|s_{t}=s,A_{t}=a\right].$$

The Q-value is updated as follows:

(11) $$Q\left(s_{t},a_{t}\right)=Q\left(s_{t},a_{t}\right)+\alpha \left[r_{t}+\gamma \max_{a}Q\left(s_{t+1},a\right)-Q\left(s_{t},a_{t}\right)\right],$$

where α denotes the learning rate. The agent’s behavior is determined based on the ϵ-greedy policy. The ϵ-greedy policy is a method of randomly selecting an action if the randomly sampled value is lower than the value of ϵ and selecting the action with the highest Q-value is greedy if it is high. However, because Q-learning uses a lookup table called a Q-table that stores Q-values in order to find the state and action pairs, it has several disadvantages: First, the probability of visiting the same state is very low. Second, a very large storage device is required to store an exponentially increasing number of state and action pairs. A DQN framework has been developed to overcome these disadvantages.

**Figure 3:**
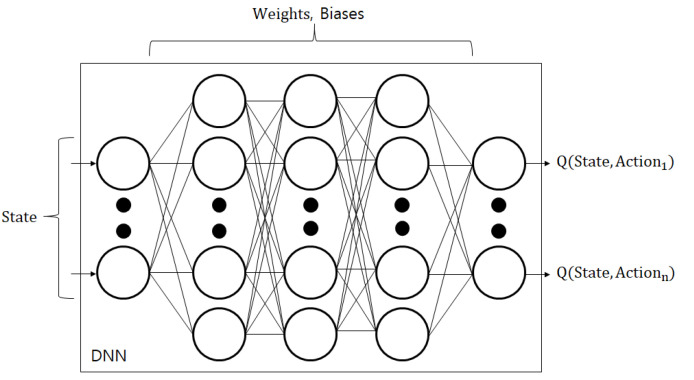
A Q-network of the DQN framework.

As shown in Figure 3, the DQN framework calculates Q-values using a Q-network in which weights and biases are stored. Therefore, when an agent needs a Q-value that matches a state, the agent puts the state as input to the Q-network and obtains the appropriate Q-value as output. The loss function for training the Q-network is as follows:

(12) $$\begin{array}{ccc} Loss(\theta ,\beta ) & = & \sum_{s_{t},a_{t}\in E}{\left(y-Q\left(s_{t},a_{t},\theta ,\beta \right)\right)}^{2} \end{array}$$

(13) $$\begin{array}{ccc} y & = & r_{t}+\gamma \max_{a}Q\left(s_{t+1},a,\theta ,\beta \right), \end{array}$$

where θ and β mean the weights and biases in the Q-network, respectively. $Q(s_{t},a_{t},\theta ,\beta )$ means the Q-value. *E* is a mini-batch sampled from the experience replay memory that stores the state, action, and reward of the next-state tuples collected while the agent interacts with the environment. However, in order for the Q-network to perform an approximation function in the DQN framework, a training process that adjusts the weights and biases stored in the Q-network is required.

### 4.3. Training and Testing Algorithm

We train the DQN with the following methods: First, we use a data sampling method with experience replay memory. Data sampling is used to remove the temporal relationships between the used data to learn the Q-network. Here, the experience replay memory is a data storage technique in which the agent collects data while interacting with the environment. The data consist of tuples of the state, action, reward, and next state. The Q-network is trained by randomly sampling data tuples from experience replay memory. In this paper, the experience replay memory is denoted by D. Second, we use a fixed target network method that includes two Q-networks, the target network and the online network, in the training process. $Q(s_{t+1},a,\theta ,\beta )$ of (13) is calculated as the target network, and $Q(s_{t},a_{t},\theta ,\beta )$ of (12) is calculated as the online network. Additionally, the weights of the online network are periodically copied to those of the target network.

The training process is described in Algorithm 1. Parameters of the online and target networks are initialized (lines 1–3) . The agent observes the state in the environment and selects the action according to the ϵ-greedy policy (lines 10–11) . That is, the agent performs a random selection with the probability of ϵ, inputs the current state to the online network with the probability of ϵ−1, and selects the largest value among the observed Q-values as output. The selected action is saved in the joint action group (line 12) . If the agent selects the actions for all V2V links, the joint action group interacts with the environment and acquires a reward (line 14) . The data tuples collected through the above process are stored in the experience replay memory (lines 15–16) . The sampled data tuples are used to update the online network. When the online network repeatedly updates the weights and biases, the weights and biases of the online network are copied to those of the target network (lines 17–25) .

The testing process is described in Algorithm 2. Unlike the training process, the testing process greedily selects an action based on the Q-network learned by the training process (lines 8–9) . After that, the action is stored in the joint action group in the same way as the training process (line 10) . If the agent selects the action for all V2V links, the joint action group interacts with the environment (line 12) . When the time step *t* reaches the simulation end time, the performances are evaluated in terms of the sum rate of the V2I and V2V links, the outage probability of V2V links, and the average power consumption of V2V links (lines 14–17) .
**Algorithm 1:** Training algorithm1:Initialize the online Q-network with random weights θ and random biases β;2:Initialize the target Q-network with random weights $\theta^{target}$ and random biases $\beta^{target}$;3:Generate Experience replay memory D;4:**for** each episode e **do**5:    Initialize environment;6:    Generate V2V and V2I networks;7:    **for** each time step *t* **do**8:          Generate a joint action group *A*;9:          **for** each V2V links **do**10:           Get state $s_{t}$ from the environment11:           Choose an action $a_{t}$ based on the ϵ-greedy policy;12:           Append the $a_{t}$ to *A*;13:        **end for**14:        Interact with the environment based on *A* and Calculate reward $r_{t}$;15:        Get all V2V links state $s_{t+1}$ from the environment;16:        Append the $s_{t}$, $a_{t}$, $r_{t}$, $s_{t+1}$ to D;17:        **for** each update step *i* **do**18:           Sample a mini-batch of experience set *E* from the D;19:           Calculate the loss:20:           *y*=$r_{t}$+γ$\max_{a}\left(Q\left(s_{t+1},a,\theta^{target},\beta^{target}\right)\right)$;21:           Loss(θ,β)=$\sum_{s_{t},a_{t}\in E}{\left(y-Q\left(s_{t},a_{t},\theta ,\beta \right)\right)}^{2}$;22:           Update the online Q-network with θ, β;23:        **end for**24:        Update weights, $\theta^{target}\leftarrow \theta$25:        Update biases with $\beta^{target}\leftarrow \beta$26:    **end for**27:**end for**

**Algorithm 2:** Testing algorithm
1:Load the Q-network with trained weights θ and biases β;2:**for** each episode e **do**3:    Initialize environment;4:    Generate V2V and V2I networks;5:    **for** each time step *t* **do**6:         Generate a joint action group *A*;7:         **for** each V2V link **do**8:              Get state $s_{t}$ from the environment9:              Choose the $a_{t}$ with the maximum value among the estimated Q-values by inputting the $s_{t}$ into the Q-network;10:            Append the $a_{t}$ to *A*;11:        **end for**12:        Interact with the environment based on *A*;13:    **end for**14:    Calculate the sum rate of V2I links;15:    Calculate the sum rate of V2V links;16:    Calculate the outage probability of V2V links;17:    Calculate the average transmit power of V2V links;18:
**end for**



## 5. Simulation Results

**Figure 4:**
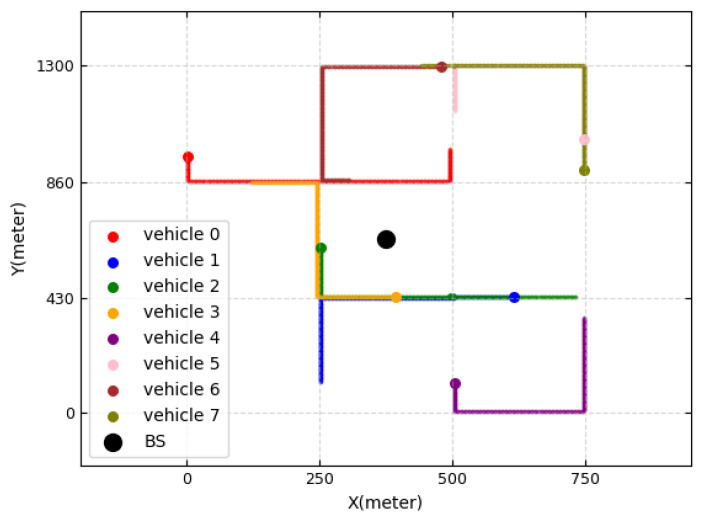
Vehicle movements.

We consider a single-cell system with one base station and 20 V2I links. We follow the simulation setup for the urban case study of Manhattan with 9 blocks of 3GPP TR 36.885 [11,40]. The models of vehicle drops, mobility, and channels all follow the evaluation scenario of 3GPP TR 36.885. Vehicles are dropped on the road according to a spatial Poisson process, and the vehicle locations are updated every one time slot in the simulation. A vehicle moves at a constant speed defined in Table 1. The vehicle changes its direction at the intersection to go straight with a probability of 0.5, to turn left with a probability of 0.25, and to turn right with a probability of 0.25. Figure 4 shows the movement of vehicles for 20 s, where there are eight vehicles and one BS. The V2V channel model and V2I channel model are both described in Table 1, according to 3GPP TR 36.885. Each vehicle communicates with a vehicle nearby. We perform a time-driven simulation, where the simulation clock is advanced in increments of time slot units and the state variables are updated for every time slot. For each slot in the simulation, we calculate the CSI of the V2I and V2V links and the interference power, which results in the state of the DQN. The simulation parameters are summarized in Table 1.

In the proposed Q-network, the number of neurons in the input layer is set to 82, the number of neurons in the hidden layers is set to [500, 250 120], and the number of neurons in the output layer is set to 60. The activation function of the hidden layers uses the ReLU function. The optimizer for training the Q-network uses RMSProp. The detailed parameters of the DQN framework are summarized in Table 2. The DQN is trained for 20,000 episodes, where an episode means 1 simulation time and new vehicles are dropped each time an episode starts. After training the DQN, the simulation is run 1000 times, and the 1000 results are averaged.

The proposed resource allocation is compared with the random resource allocation and the conventional RL-based resource allocation of [11] in terms of the average transmit power of the V2V links, the average outage probability of the V2V links, and the average sum rates of the V2V and V2I networks. In the random resource allocation, the transmitter of the V2V link transmits data with randomly selected transmit power through a randomly selected RB.

**Figure 5:**
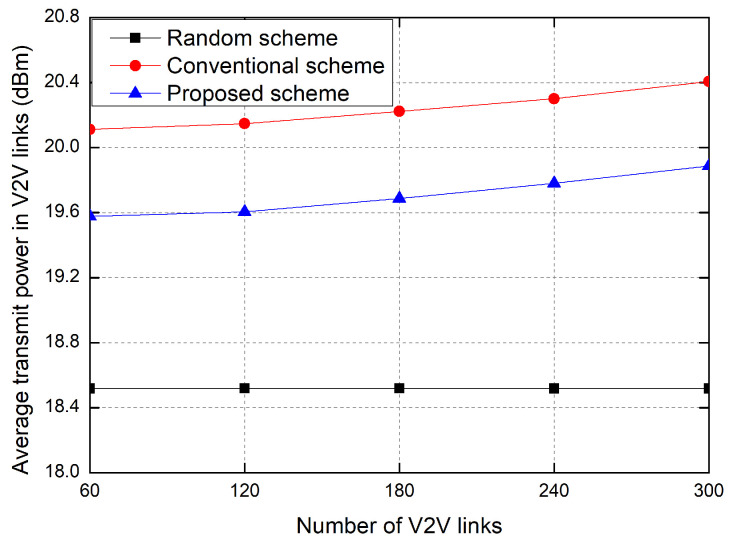
Average transmit power of V2V links.

Figure 5 shows the average transmit power of V2V links according to the number of V2V links. As the number of V2V links increases, the average transmit power of vehicles increases in the proposed scheme and the conventional RL-based scheme, but the average transmit power in the random allocation scheme is fixed. Because the interference increases according to the increase in the number of V2V links, the transmit power of vehicles increases in order to overcome the interference, in the proposed scheme and the conventional RL-based scheme. The proposed scheme significantly reduces the power consumption of V2V links in comparison with the conventional RL-based scheme because of the penalty function of the transmit power in the reward. In the random allocation scheme, because the transmitter randomly selects the transmit power, the transmit power of the V2V link is fixed on average, regardless of the amount of interference caused by other V2V links. In the simulation environment of this paper, the random allocation scheme shows a low power consumption due to the low transmit power but shows an outage probability that is too high.

**Figure 6:**
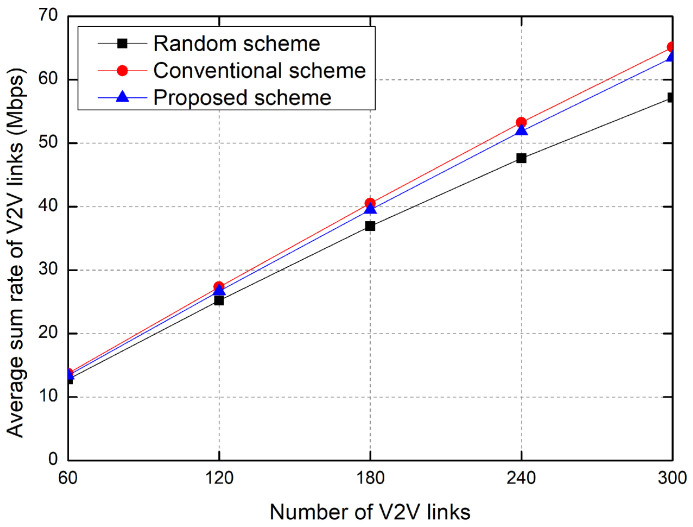
Average sum rate of V2V links.

Figure 6 shows the sum rate of V2V links as the number of V2V links increases. As the number of V2V links increases, the sum rate of V2V links increases in all the resource allocation schemes. The sum rate of the conventional RL-based scheme is slightly higher than that of the proposed scheme because the proposed scheme suppresses the transmit power of vehicles for the purpose of energy efficiency. The sum rate of the random allocation scheme is the worst because it randomly selects the RBs regardless of the interference to others.

**Figure 7:**
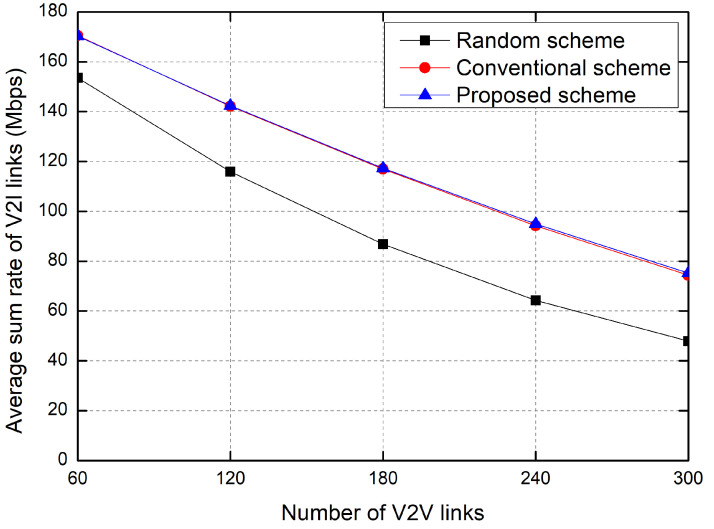
Average sum rate of V2I links.

Figure 7 shows the sum rate of V2I links as the number of V2V links increases. Because the number of V2I links is fixed at 20, the interference from the V2V links increases according to the number of V2V links, and thus the sum rate of V2I links decreases with the increase in the V2V links. In particular, the performance of the proposed scheme is slightly better than that of the conventional RL-based scheme. Moreover, because the interference from the V2V links to the V2I link increases according to the number of V2V links, the sum rate of the random allocation greatly decreases with the number of V2V links.

**Figure 8:**
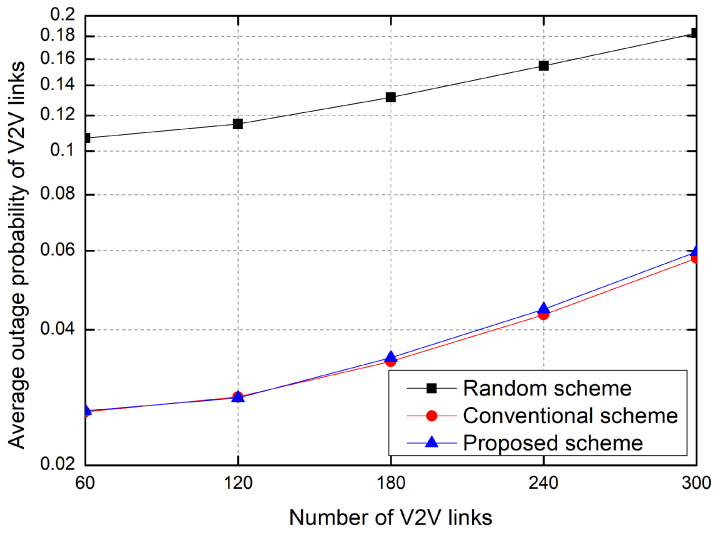
Average outage probability of V2V links.

Figure 8 shows the outage probability as the number of V2V links increases. Here, the outage probability is defined as the probability that a transmitter on the V2V link fails to transmit data within the maximum allowable latency, $T_{0}$. The outage probability is inversely proportional to the sum rate. Hence, the outage probability increases according to the number of V2V links. Moreover, the outage probability of the random allocation scheme is much higher than that of other schemes. That is, in order to efficiently allocate resources, RL-based resource allocation is required.

The major concerns with deep-learning-based approaches are the computational complexity and the memory space, which depend on the number of parameters to be stored and to be computed. In the proposed RL, from (8), the dimension of the state space is $D_{\mathrm{state}}=\left(4\times N_{\mathrm{RB}}\right)+2$ (=82 in our simulation) and the dimension of the action space is $D_{\mathrm{action}}=N_{\mathrm{pwr}}\times N_{\mathrm{RB}}$ (=60 in our simulation), where $N_{\mathrm{RB}}$ is the number of RBs and $N_{\mathrm{pwr}}$ is the number of the transmit power levels. Consider a feed-forward network with *l* layers, where layer 0 is the input layer and layer l−1 is the output layer. Let the number of neurons of each layer be $n_{0},n_{1},\ldots ,n_{l-1}$. Then, the number of parameters (weights) of the network, including biases, is given by $N_{\mathrm{DNN}}=\sum_{i=0}^{l-2}n_{i}n_{i+1}+\sum_{i=1}^{l-1}n_{i}$ (=204,130 in our simulation). Because the DQN framework calculates Q-values using a DNN, the total number of parameters becomes $D_{\mathrm{state}}+D_{\mathrm{action}}+N_{\mathrm{DNN}}$. Moreover, because of the use of two Q-networks, the online network and the target network, the total number of parameters to be processed doubles, and the replay memory is required to store a collection of experience tuples, i.e., the parameters of the online network. In our simulation, we set the replay buffer size to 100 tuples. The computational complexity is similar to [11]. In our implementation, each selection takes less than $10^{-4}$ s using GPU 2080 Ti. The computational speed is acceptable for vehicles thanks to the power of the GPU. The computational complexity of the DNN can be reduced by using lightweight DNNs [42,43].

## 6. Conclusions

Vehicular communications or V2X are key to the development of autonomous vehicles. In the V2X network, it is important to manage radio resources efficiently to provide low-latency and energy-efficient services. In this paper, we developed a DQN-based energy-efficient resource allocation scheme in a V2X communication network in which V2I and V2V networks share resource blocks. We formulated the reward of the DQN model by using two penalties and two positives. Here, the two penalties are the transmission time and the transmit power, and the two positives are the sum rate of the V2I and V2V networks. The proposed scheme significantly reduces the power consumption of vehicles in the V2V network without sacrificing the sum rate and outage probability. The results show that an energy-efficient resource allocation scheme is crucial in order to meet the latency and power consumption requirements of mission critical V2V applications. 

## Figures and Tables

**Table 1:** Simulation parameters.

Parameter	Value
Road intersection size	430m×250 m
Simulation area size	1300m×750 m
Absolute vehicle speed	36 km/h
Vehicle drop and mobility model	Urban case of A.12 in 3GPP TR 36.885 [40]
V2V path loss model	WINNER + B1 Manhattan [41]
V2V shadowing	Log-normal with $\sigma^{2}=3$ dB
V2I path loss model	128.1 + 37.6 log(*R*), where *R* in kilometers
V2I shadowing	Log-normal with $\sigma^{2}=8$ dB
V2V and V2I fast fading	Rayleigh fading
Noise power	−114 dBm
Carrier frequency, $f_{c}$	2 GHz
Sub-carrier frequency	1.5 MHz
Number of V2I links, *L*	20
Number of V2V links, *K*,	[60, 120, 180, 240, 300]
Antenna height of eNode B type RSU	25 m
Antenna gain of RSU	8 dBi
Noise figure of RSU’s antenna	5 dB
Antenna height of vehicles	1.5 m
Antenna gain of vehicles	3 dBi
Noise figure of vehicle’s antenna	9 dB
Latency constraints for V2V link	100 ms
V2V payload size	30 Mbits
Update time slot duration	2 ms
Simulation time	400 ms
transmit power level of V2V links	[5, 10, 23] dBm

**Table 2:** DQN framework parameters.

Parameter	Value
Number of neurons in the input layer	82
Number of neurons in each hidden layer	500, 250, 120
Number of neurons in the output layer	60
Reward discount factor	0.99
Hidden layer activation function	ReLU
Optimizer	RMSProp
Learning rate α	0.001
Values of $\lambda_{V2I}$, $\lambda_{V2V}$, $\lambda_{\mathrm{Latency}}$, and $\lambda_{\mathrm{pwr}}$	0.1, 0.9, 1, and 0.2, respectively
